# Pregabalin‐Induced Myoclonus in a Patient Without Kidney Dysfunction

**DOI:** 10.1155/crnm/8902603

**Published:** 2026-06-28

**Authors:** Sokena Zaidi, Patrick D. Lyden

**Affiliations:** ^1^ Department of Neurology, Keck School of Medicine, 1540 Alcazar Street Suite 215, Los Angeles, 90033, California, USA, usc.edu; ^2^ Department of Physiology and Neuroscience, Zilkha Neurogenetic Institute of the Keck School of Medicine of USC, 1501 San Pablo Ave ZNI 245, Los Angeles, 90089, California, USA

**Keywords:** nephrotoxicity, positive myoclonus, pregabalin

## Abstract

Pregabalin is FDA‐approved for the treatment of neuropathic pain associated with diabetes, postherpetic neuralgia, spinal cord injury, and as an adjunctive treatment for partial seizures and thus is a commonly prescribed agent in the primary care, neurological, and pain management settings. Adverse effects of pregabalin include dizziness, somnolence, diplopia, blurry vision, and respiratory depression, as well as delayed dermatological hypersensitivity reactions and weight gain. Pregabalin dosing is influenced by renal function, and dose adjustment should be considered in all patients. A much rarer but serious adverse effect of pregabalin is myoclonus. There have been a few case reports of negative myoclonus in patients treated with both pregabalin and gabapentin; however, these patients tend to have renal dysfunction and typically present with altered mentation. Furthermore, these patients are often treated in the context of refractory epilepsy and are treated with other antiepileptic drugs, some of which can also result in myoclonus. Brief episodes of confusion, diplopia, word‐finding difficulty, or other transient neurological deficits, which could be concerning for acute ischemic events in the setting of pregabalin use, have never been described. Here, we present a case report of a patient on long‐term pregabalin without renal dysfunction who initially presented as a stroke alert for transient neurological deficits followed by jerking movements consistent with positive myoclonus, which is much less commonly reported than negative myoclonus in the setting of pregabalin usage.

## 1. Introduction

Pregabalin is an FDA‐approved drug for the treatment of neuropathic pain, postherpetic neuralgia, spinal cord injury, and as an adjunctive treatment for partial seizures and thus is a commonly prescribed agent in the primary care, neurological, and pain management settings [1]. The mechanism of action for pregabalin is similar to gabapentin, and it binds the alpha‐2‐delta subunit of voltage‐gated calcium channels, regulates calcium transport, and subsequently modulates the release of glutamate, noradrenaline, and substance P, overall inhibiting neuronal excitation [2]. Pregabalin is excreted exclusively renally; thus, renal dosing is of paramount importance in patients. It is not a CYP inhibitor or inducer and is therefore not commonly implicated in drug–drug interactions [3]. The most common adverse effects of pregabalin are primarily neurological, and in randomized control trials, dizziness, somnolence, and ataxia appear to be most frequently encountered [4], followed by blurry vision, diplopia, tremors, and impaired concentration, with the most severe adverse effect being respiratory depression [5]. Dosing starts at 150 mg of pregabalin divided over 2–3 doses, with a slow titration schedule and close monitoring of symptoms [6]. A much rarer but profound adverse effect of pregabalin is myoclonus, which has been reported primarily in patients with either renal dysfunction or in patients already on other antiepileptic drugs that also carry a risk for myoclonus [6]. Interestingly, brief episodes of confusion, diplopia, word‐finding difficulty, or other transient neurological deficits, which were concerning for acute ischemic events, have never been described. Myoclonus can be either “positive” or “negative,” with positive myoclonus referring to brief muscular contractions, whereas negative myoclonus refers to direct inhibition of ongoing muscle contractions [7].

## 2. Case Report/Case Presentation

A 64‐year‐old female with known L4–L5 spondylolisthesis and known C5–C6 disc degeneration pending planned neurosurgical intervention presented as a stroke code alert to the emergency department at Los Angeles General Medical Center for an acute episode of word‐finding difficulty, which had resolved at the time of stroke team evaluation.

Her lumbar spinal stenosis presented with bilateral buttock pain without any weakness, which had been ongoing for at least 1 year. She had already been evaluated by neurosurgery at an outside hospital and was found to have symptomatic mobile spondylolisthesis at L4–L5, as well as moderate to severe cord compression at C5–C6 in the setting of multilevel disc degeneration and severe spinal canal stenosis with moderate bilateral neural foramina narrowing. She was pending likely anterior cervical discectomy and fusion of C5–C6, followed by lumbar spine neurosurgical intervention. She had been managing her pain with the following regimen: pregabalin 200 mg nightly, venlafaxine 150 mg nightly, bupropion 300 mg nightly, and baclofen 5 mg nightly as needed. According to the patient, as confirmed by review of pharmacy records, it appears that her pharmacy dispensed a 300‐mg capsule to the patient, whereas previously she was taking eight 25 mg tablets of pregabalin for a total of 200 mg nightly as her regimen. The patient had never exceeded the 200‐mg dose for nightly pregabalin. Notably, the patient denied any history of fevers, chills, night sweats, or B symptoms pointing to underlying malignancy, and she did not endorse any family history of neoplasm.

She went to sleep in her usual state of health the night prior at 8:30 p.m., was ambulatory, with no speech difficulties or neurologic deficits. She awoke the next morning at 8:30 a.m. with horizontal double vision in both eyes, subjective brain fog, and an episode of difficulty speaking, which began acutely and self‐resolved in less than 1 min. After this, she immediately noticed tremors of her entire body as well as uncontrollable jerking. The patient and her husband endorsed intermittent multifocal limb jerking lasting less than 5 s, as well as intermittent isolated extremity jerking, which was nonsustained and less than 5 s. They denied any loss of tone. During the entire course, she did not have any loss of consciousness, headache, confusion, gaze deviation, slurred speech, further vision changes, or a postictal period. She also denied any sensory or motor deficits and was able to ambulate in the emergency department. On arrival, she was afebrile, normotensive, with a heart rate in the 80s and a blood sugar in the 80s, with an NIHSS score of 0. A CT head was obtained, which did not show any hypodensities, midline shift, or loss of gray–white differentiation. Vessel imaging was deferred as she had a nonfocal examination and no signs or symptoms suggestive of large vessel occlusion. EKG showed normal sinus rhythm, QTC of 430, a PR interval of 143. While she was in the CT scanner, she had episodes of full‐body jerking, as well as intermittent isolated arm and leg jerking, which were nonsustained.

We observed brief intermittent nonrhythmic jerking consisting of increased tone with relaxation afterward, sometimes originating in the neck, sometimes originating in a singular extremity, and sometimes in multiple limbs at once, without loss of consciousness. After review of these jerks, we determined that the patient had positive myoclonus because of ongoing brief muscular contractions, versus direct inhibition of ongoing muscle contractions, which is seen in negative myoclonus [7]. The patient retained her ability to converse throughout. No gaze deviation was observed at any time. In terms of laboratory evaluation, the CBC was unremarkable, and the BMP was unremarkable with the exception of calcium at 10.4. Notably, her creatinine level was 0.86 mg/dL, with a calculated GFR of 75 mL/min. Urinalysis was unremarkable, and the urine drug screen was negative. Ethanol, salicylate, and acetaminophen levels were within normal limits.

In the emergency department, she was provided with gentle reassurance, treated with 1 L normal saline, and 1 mg oral Ativan followed by 1 mg intravenous Ativan followed by complete resolution of all jerking events and symptoms. The patient was observed for 6 h in the emergency department and returned to her baseline, was ambulatory, and felt well enough to leave the hospital. She was recommended to follow up with her primary care physician.

## 3. Discussion

We report a case of a patient with normal kidney function presenting with transient word‐finding difficulties, diplopia, confusion, and myoclonic jerks concerning for an acute ischemic event after an inadvertent increase in pregabalin dose.

Myoclonus is characterized by sudden, brief muscle jerks, which are involuntary in nature. It can be further classified as positive myoclonus, which refers to abrupt muscle contraction, and negative myoclonus, which refers to sudden termination of muscular activity [8]. Our case satisfied the definition of positive myoclonus. Myoclonus secondary to antiepileptic drugs including pregabalin, gabapentin, phenytoin, carbamazepine, oxcarbazepine, lamotrigine, vigabatrin, and tiagabine has been described; however, these are more often seen in patients with known progressive myoclonic epilepsy syndromes [9]. Huppertz et al. describe a series of four patients with medically refractory focal epilepsy who were newly treated with pregabalin in the context of already receiving other antiepileptic agents and subsequently developed myoclonus [3]. Kidney function was not reported for any of these patients, and no other focal neurologic findings were noted. For three of these patients, myoclonus was subtle and did not interfere with their daily activities. Knake et al. describe two case reports of patients with mild kidney dysfunction, presenting with myoclonus after initiating pregabalin for chronic pain, with EEG correlate of polyspike wave complexes, which resolved with IV lorazepam administration [10]. Hellwig et al. describe a case of a patient with significant renal dysfunction, exhibiting negative myoclonus and inability to ambulate in the setting of a single 100‐mg pregabalin administration, which disappeared after administration of 2 mg IV clonazepam and discontinuation of pregabalin [11]. Kim et al. found nine cases of negative myoclonus induced by pregabalin; however, they did not identify any cases of positive myoclonus [12]. Interestingly, these patients all developed negative myoclonus within 4 days of administration of pregabalin.

This present case report highlights a rare but significant adverse effect of pregabalin: word‐finding difficulty, confusion, diplopia, and persistent positive myoclonus in a patient without renal dysfunction on long‐term pregabalin for pain management with a singular dose increase of 100 mg. The transient neurological deficits have not been previously described in the context of pregabalin use. Furthermore, negative myoclonus is more classically associated with pregabalin use and in the context of other antiepileptic drug usage. This case suggests that pregabalin can cause acute neurological symptoms and positive myoclonus even in patients without renal impairment, particularly with dosing errors. Our case further underscores the critical importance of a thorough medication history as possible during the time constraints of a code stroke.

## Author Contributions

Sokena Zaidi contributed to the identification of the case, drafting of the manuscript, finalization, and submission. Patrick D. Lyden contributed to securing funding and revising the manuscript.

## Funding

This study was supported by grant U24 NS130600 [Patrick D. Lyden].

## Ethics Statement

This work was approved via exemption from informed consent by the IRB at the Keck School of Medicine of USC.

## Conflicts of Interest

The authors declare no conflicts of interest.

## Data Availability

No data were generated in this study.
